# Pituitary apoplexy presenting with acute kidney injury

**DOI:** 10.1186/s13256-023-04019-4

**Published:** 2023-06-13

**Authors:** Ahmet Murt

**Affiliations:** 1 Nephrology Clinic, Bingol State Hospital, Bingol, Turkey; 2 Istanbul University-Cerrahpasa, Cerrahpasa Medical Faculty, Istanbul, Turkey

**Keywords:** Pituitary apoplexy, Acute kidney injury, AKI

Dear Editor,

I read the case report by Abo, KM et al. of a 76-year-old patient with postoperative pituitary apoplexy with great interest. As underlined by the authors, intraoperative blood loss may be an additional risk factor for pituitary ischemia, infarction, and hemorrhage in patients who have pituitary adenoma [1]. The patient presented by Abo, KM et al. had partial right nephrectomy. Pituitary apoplexy (PA) as well as acute kidney injury (AKI) with anuria in that patient were attributed to the intraoperative blood loss.

Although AKI may be a consequence of partial nephrectomy and blood loss in that patient, I would like to point out PA as a possible cause of AKI. Among hormone deficiencies related to PA, cortisol deficiency is particularly important as its consequences such as hypotension and hyponatremia might be life threatening. In addition, as a result of hemodynamic instability in hypotensive state, acute kidney injury (AKI) may be observed. I have recently encountered a patient with PA who presented with AKI.

Similar to the presented case, he was also 76 years old. In previous medical history, he only had hypertension and was stable on olmesartan and hydrochlorothiazide treatment. He experienced occasional headache and vomiting for the last month and applied to another clinic for his symptoms 1 week ago. According to electronic health records, his neurologic examination was unremarkable and his blood pressure was 135/90 mmHg then. On laboratory tests, creatinine level was 1.35 mg/dL [normal range (NR) 0.7–1.2 mg/dL]. He was prescribed paracetamol and invited for a control visit. In the control visit 1 week later, his general health state was worse with reduced consciousness. Laboratory tests revealed increased creatinine levels of 4.00 mg/dL, and he was referred to our clinic for a nephrology consultation. On physical examination he was hypovolemic and his blood pressure was 110/70 mmHg. His heart rate was 88 beats per minute in sinus rhythm, and Glasgow Coma Scale score was 13. The patient was admitted to the nephrology ward with AKI. A cranial computed tomography (CT) was ordered because of the persistence of headache with reduced consciousness. CT images raised the suspicion of sellar enlargement with soft tissue densities in the sella. Hypophysis magnetic resonance imaging (MRI) confirmed a pituitary adenoma. He was diagnosed with PA, and 40 mg methylprednisolone was initiated with relevant concomitant fluid therapies. Creatinine levels gradually decreased to 1.56 mg/dL in a week, and clinical symptoms of the patient improved. Thyroid-stimulating hormone (TSH) (0.157 mU/L; NR 0.27–4.20 mU/L) and thyroxine (0.905 ng/dL; NR 0.93–1.70 ng/dL) levels were found to be decreased, and levothyroxine supplementation was also initiated. The following other pituitary hormone levels were checked in our clinic: prolactin 41.39 μg/L (NR 4.04–15.2 μg/L), adrenocorticotropic hormone (ACTH) 7.52 ng/L (NR 0–46 ng/L), follicle-stimulating hormone (FSH) 8.0 IU/L (NR 1.5–12.4 IU/L). His morning cortisol level was 7.06 μg/dL (NR 4.8–19.5 μg/dL), and somatomedin-C level was 51.3 ng/mL (NR 59–177 ng/mL). The patient was discharged with endocrinology and neurosurgery control referrals.

With this case, I would like to underline that PA may also present with AKI. AKI is defined as an absolute 0.3 mg/dL increase in creatinine levels in the last 48 hours or an increase to 1.5 times of its basal level in the last 7 days [2]. Acute kidney injury may be due to hypotension in patients with PA. Volume depletion because of excessive vomiting and nonsteroidal anti-inflammatory drugs that are prescribed for headache are also risk factors for AKI. While reports of PA cases with AKI are not many [3], the clinical course may be so severe that even hemodialysis may be required [4].

In conclusion, AKI may be one of the rare clinical presentations of PA, and renal function should be regularly monitored in patients with PA.

## Data Availability

The data presented in this letter are available from the corresponding author upon reasonable request.
